# Enhancing Self-care Among Oral Cancer Survivors: Protocol for the Empowered Survivor Trial

**DOI:** 10.2196/39996

**Published:** 2023-01-20

**Authors:** Sharon L Manne, Matin Imanguli, Deborah Kashy, Morgan Pesanelli, Sara Frederick, Janet H Van Cleave, Lisa Paddock, Shawna Hudson, Michael Steinberg, Patrick Clifford, Mara Domider, Neetu Singh

**Affiliations:** 1 Division of Medical Oncology Rutgers Cancer Institute of New Jersey New Brunswick, NJ United States; 2 Division of Head and Neck Oncologic and Reconstructive Surgery Rutgers Robert Wood Johnson Medical School New Brunswick, NJ United States; 3 College of Social Science Department of Psychology Michigan State University Lansing, MI United States; 4 School of Public Health Rutgers State University of New Jersey Piscataway, NJ United States; 5 Rory Meyers College of Nursing New York University New York, NY United States; 6 Rutgers Cancer Institute of New Jersey New Brunswick, NJ United States; 7 Department of Family Medicine and Community Health Institute for Health Sciences Rutgers Robert Wood Johnson Medical School New Brunswick, NJ United States; 8 Department of General Internal Medicine Rutgers Robert Wood Johnson Medical School New Brunswick, NJ United States; 9 Department of Health Behavior, Society, and Policy School of Public Health Rutgers State University of New Jersey Piscataway, NJ United States

**Keywords:** oral cancer, cancer survivorship, quality of life, digital interventions

## Abstract

**Background:**

Survivors of oral cavity and oropharyngeal cancer frequently experience difficulties in swallowing; tasting; speaking; chewing; and maintaining comfortable movements of the head, neck, and shoulder. Engagement in regular self-care can reduce further loss of function and mitigate late effects. Despite the substantial self-care requirements, there are no empirically based interventions to enhance the skills and confidence of these survivors in managing their ongoing care.

**Objective:**

The aim of this study is to describe the rationale and methodology for a randomized controlled trial evaluating Empowered Survivor (ES) versus Springboard Beyond Cancer, a general web-based program for cancer survivors, on self-efficacy in managing care, preparedness for managing survivorship, and health-related quality of life (QOL).

**Methods:**

This study will recruit a total of 600 individuals who were diagnosed with oral cavity or oropharyngeal cancer in the past 3 years and are currently cancer free primarily from state cancer registries; these individuals will be randomly assigned to either the ES or Springboard Beyond Cancer condition. The participants complete measures of self-efficacy in managing care, preparedness for survivorship, health-related QOL, and engagement in oral self-examination and head and neck strengthening and flexibility exercises at baseline and 2 and 6 months after baseline. The primary aim of this study is to evaluate the impact of ES versus Springboard Beyond Cancer on self-efficacy, preparedness, and health-related QOL. The secondary aim is to examine the mediators and moderators of ES’s impact on self-efficacy in managing care, preparedness, and health-related QOL at 6 months. The exploratory aim is to conduct a process evaluation of ES to identify potential oncology or community settings for future implementation.

**Results:**

Multilevel modeling will be used to examine whether there are significant differences between the ES and Springboard Beyond Cancer interventions over time. Mediational models will evaluate the indirect effects of ES on outcomes. Quantitative analyses will evaluate the predictors of ES use, and qualitative analyses will evaluate the preferred timing and settings for the implementation of ES.

**Conclusions:**

This randomized controlled trial evaluates a completely web-based intervention, ES, versus a general web-based program for cancer survivors, Springboard Beyond Cancer, on self-efficacy in managing care, preparedness for managing survivorship, and health-related QOL and identifies the putative mediators and moderators of the intervention’s effects. If an effect on the primary outcomes is illustrated, the next step could be an implementation trial to evaluate the intervention’s uptake in and impact on an oncology care setting or nonprofit organizations.

**Trial Registration:**

ClincalTrials.gov NCT04713449; https://clinicaltrials.gov/ct2/show/NCT04713449

**International Registered Report Identifier (IRRID):**

DERR1-10.2196/39996

## Introduction

### Background

In the United States, the American Cancer Society (ACS) estimates that there will be 54,000 new oral and oropharyngeal cancer cases, which will result in 11,230 deaths, in 2022 [1]. There are approximately 91,000 people living with oral cancer in the United States [1]; 50% of these are diagnosed with regional disease, and 17% are diagnosed with distant disease. Traditionally, the main risk factors for oral cancer have been tobacco and alcohol consumption [2]. The incidence also increases with age, with >90% of oral cancer cases occurring in individuals aged >45 years [3]. Gastroesophageal reflux disease has been associated with oral cancer [4]. Human papilloma virus (HPV) has been associated with oropharyngeal cancer and few other oral cancers [5]. This patient population differs from the population with non-HPV–associated oral cancers in that they are primarily White, nonsmoking, nondrinking males aged between 35 and 55 years who do not have other health issues [6,7]. The 5-year survival rate for all forms of oral cancer has increased from 52.5% to 66.3% over the past 30 years [8]. However, patients with HPV-associated oropharyngeal cancers have a much higher survival rate (80% at 5 years) and longer disease-free survival rate, largely because they are more responsive to therapy [9,10]. There are known disparities in oral cancers. Mortality from oral cancers is higher among Black men [11-15], among men in general [16], and increasing among individuals aged between 55 and 64 and those aged between 65 and 74 years [3,17,18]. In summary, there is a growing population of oral and oropharyngeal cancer survivors, particularly those with HPV-associated cancer [3].

Oral and oropharyngeal cancer survivors experience challenges in both the treatment regimen and long-term self-management and surveillance needs. Treatment can entail surgery, chemotherapy, or radiation. Because of the location of the structures involved, the disease, and the treatment, this cancer can result in debilitating and permanent disfigurement and functional changes that interfere with the ability to swallow, taste, speak, eat, and move the shoulders and neck [19-23]. Long-term medical maintenance needs are complex and can require a diverse care team that can include oncologists, reconstructive surgeons, dentists, physical rehabilitation specialists, speech and swallowing experts, and nutritionists to restore functions or prevent the deterioration of functions [24-27]. Current models of survivorship care place most of the day-to-day responsibility on self-management and care coordination. Even with rehabilitation, many survivors recover their precancer functioning. Thus, the primary goal is to prevent the deterioration of function and maintain comfort. These self-care needs persist throughout the survivor’s lifetime.

Adding to these self-management needs are surveillance expectations. Because 20% of survivors develop a recurrence or metastatic disease within the first 2 years after treatment [28], the recommended follow-up regimen includes frequent surveillance by the oncology care team and survivors [1,29,30]. Regular self-examinations to locate suspicious areas in the mouth and neck are recommended [31], as lesions that are not evident on positron emission tomography scans can be detected earlier and checked by a professional. Early detection can reduce morbidity and mortality [32,33]. Adherence to this demanding care regimen is less than optimal: 30% to 50% do not adhere to follow-up appointments, and up to 80% do not adhere to oral care or swallowing recommendations [34-37]. More than half of the survivors continue to drink alcohol, which increases the risk of mortality [38].

This cancer can result in a deterioration in health-related quality of life (QOL). Both cross-sectional studies conducted over the survivorship trajectory [39] and longitudinal studies suggest that patients experience lower health-related QOL [40]. Cross-sectional studies have indicated that global QOL, oral function (chewing, swallowing, tasting, salivating, and speaking), dental issues, and appearance are low over the decade after diagnosis, and studies illustrated higher levels of depression and anxiety (Rogers et al [41]) [41-53]. Longitudinal studies have had variable findings depending on sample composition, time of assessments, and time since diagnosis. As a whole, results suggest that some domains of QOL improve over time (eg, depression, anxiety, appearance, speech, and pain), some return to baseline levels (leisure time activities and swallowing), but many deteriorate or continue to illustrate decrements (eg, dry mouth, shoulder mobility, taste, problems with teeth, sexuality, and role function for work and leisure activities) [54-56]. The literature consistently suggests that survivors report persistent support needs, which include the desire for more information and support regarding long-term effects, recommended follow-up, monitoring symptoms that prompt contacting a physician, maintaining nutrition, improving speech and swallowing, maintaining oral health, and addressing psychological concerns [57-63]. The fact that more than half of the survivors do not receive a survivorship care plan complicates matters and may reduce their ability to manage their posttreatment care [64-67]. Due to the ongoing care needs and compromised QOL, it is not surprising that survivors report low self-efficacy in managing their self-care regimen, particularly with regard to conducting oral self-examinations, maintaining proper nutrition, and managing dry mouth and swallowing issues [65,66,68].

Effective self-management provides survivors with the ability to monitor their cancer-related symptoms and self-care and implement practices needed to maintain satisfactory functioning [69]. Despite the substantial care needs, low self-care self-efficacy, and low participation in self-care, there are only a few self-management interventions for this population. To date, the few published studies have focused on single-symptom pilot studies (eg, swallowing or couple communication) [65,70-72]. To address the need for improved self-management and bolster the self-efficacy in managing self-care, we developed and evaluated the feasibility and preliminary efficacy of a web-based self-management intervention for oral cancer survivors’ care called Empowered Survivor (ES).

### Theoretical Framework

The development of the ES intervention is guided by the theoretical construct of self-efficacy, which is drawn from social cognitive theory [73,74]. Social cognitive theory focuses on the development of competencies and self-regulation [73,74]. Self-efficacy is a competency defined as the belief in one’s ability to execute actions to deal with a situation successfully and comprises 3 domains: knowledge and skills to accomplish the task, confidence in one’s ability to motivate oneself and the resources available to accomplish the task, and confidence in one’s ability to execute the task [75]. Self-efficacy is a task-specific expectation: people estimate their confidence in their ability to manage a task by evaluating the tasks involved in the successful completion of that task [73,74]. Individuals with high self-efficacy beliefs and abilities regarding a task call upon themselves to execute the task. Confidence in one’s ability to execute a task is the most widely studied dimension of self-efficacy. Self-efficacy is linked to the initiation and maintenance of health behaviors [75-83], and greater efficacy is associated with better health behaviors among cancer survivors [84]. Efficacy also predicts psychosocial and functional outcomes among cancer survivors [85,86]. Social cognitive theory has formed the basis of effective intervention programs for improving health behaviors [87-89], disease management among chronically ill individuals, and [90] the health and well-being of cancer survivors [91].

**Figure 1:**
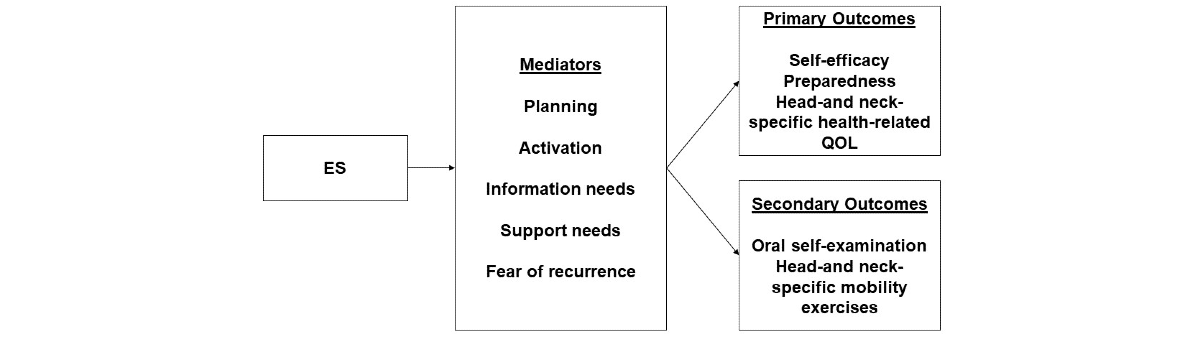
Heuristic framework for Empowered Survivor (ES). QOL: quality of life.

Bolstering self-efficacy in survivorship care is a primary outcome and target of ES. To delineate the mediators of ES’s impact, we propose a heuristic framework based on social cognitive theory. First, we use the work of Gollwitzer and Schwarzer, which proposes that planning is a key component of self-management and goal achievement [81,92-94]. In our pilot study, planning was associated with greater efficacy and survivorship preparedness [65]. Second, we integrate the construct of patient activation, defined as the willingness to take actions to manage care and understanding one’s role in survivorship care [95]. Greater activation is associated with higher self-efficacy in our study [65] and is targeted by ES. Third, we include information needs, defined as the knowledge about treatment effects and care responsibilities. Knowledge of tasks improves self-efficacy, and lower information needs are associated with higher self-efficacy. Fourth, we include support needs, defined as the level of assistance required to accomplish self-care tasks. Unmet support needs are strongly linked to lower self-efficacy [65]. Finally, the fear of cancer recurrence is a common issue among oral cancer survivors and is associated with poorer QOL and lower self-efficacy [49,96,97]. Reducing the fear of recurrence by teaching coping strategies should increase self-efficacy. Our heuristic framework is illustrated in Figure 1. We have selected 3 long-term outcomes to reflect the key components of survivorship self-management: self-efficacy to manage care, preparedness for survivorship, and head- and neck-specific QOL. Finally, the role of self-management interventions in improving the engagement in self-management tasks among survivors has not been well studied. There is extensive literature suggesting that self-efficacy interventions have an impact on self-management among adults with chronic illness [98-101]. This study explores the impact of ES on self-management behaviors. We chose self-management behaviors that are commonly recommended for oral cancer survivors: oral self-examinations and head and neck mobility exercises. Self-examinations assist survivors in finding suspicious lesions early, and head and neck mobility exercises maintain the ability to easily eat, speak, and move. Engagement in both is low. There are other self-management behaviors (eg, not drinking alcohol and not smoking), but they are not issues for all survivors.

### Study Objectives

#### Overview

The purpose of this study is to evaluate the efficacy of a web-based intervention, ES, against a free, publicly available web-based self-management intervention developed for cancer survivors by the National Cancer Institute (NCI) and the ACS called Springboard Beyond Cancer [102]. A total of 600 patients diagnosed with a first primary oral or oropharyngeal cancer within the past 3 years are being recruited via the New Jersey State Cancer Registry (NJSCR), Cancer Registry of Greater California, and Pennsylvania State Registry and randomly assigned to ES or Springboard Beyond Cancer. The participants complete measures at baseline and at 2 and 6 months after baseline. A process evaluation of ES is completed. The study has 3 primary aims and 1 exploratory aim.

#### Primary Aim 1

The first primary aim is to evaluate the impact of ES versus Springboard Beyond Cancer on the primary outcomes of self-efficacy in managing care, preparedness for managing survivorship, and head- and neck-specific QOL. We hypothesize that the participants randomized to ES will report significantly greater improvements in self-efficacy for managing their care, preparedness for survivorship, and head- and neck-specific QOL at 6 months.

#### Primary Aim 2

The second primary aim is to evaluate the mediators (planning, information needs, support needs, activation, and fear of recurrence) and moderators (age, race and ethnicity, HPV status, baseline self-efficacy, and preparedness) of ES’s impact on self-efficacy in managing care, preparedness, and health-related QOL. The primary outcomes are self-efficacy in managing self-care, preparedness for managing survivorship, and health-related QOL at 6 months. We hypothesize that the impact of ES on self-efficacy, preparedness, and health-related QOL will be mediated by improvements in survivorship care planning, reductions in information and support needs, and reductions in the fear of recurrence. Little is known about which survivor subgroups benefit the most from self-management interventions. In addition, we hypothesize that the survivors with fewer resources (survivors who belong to people of color, with lower education, who have not received a written survivorship care plan, with less baseline self-efficacy, and with less baseline preparedness) will benefit more from ES.

#### Primary Aim 3

The third primary aim is to evaluate the impact of ES versus Springboard Beyond Cancer on the secondary outcomes of engagement in oral self-examinations and head and neck mobility exercises.

We hypothesize that the participants randomized to ES will report more frequent engagement in and comprehensiveness of oral self-examinations and head and neck mobility exercises than the participants randomized to Springboard Beyond Cancer at 6 months.

#### Exploratory Aim

The exploratory aim is to conduct a process evaluation of ES to inform future implementations.

We hypothesize that the participants randomized to ES will use the intervention more, evaluate it more highly, have greater improvements in self-efficacy, preparedness, and QOL.

## Methods

### Ethics Approval

This study was reviewed and the protocol procedures were approved by the Rutgers University Institutional Review Board (protocol number Pro2020000768). All the participants had a discussion with a member of the research team and read and acknowledged electronic informed consent by clicking “agree to participate” before completing the baseline survey. The participants are assigned a study ID upon entry, and data are stored under the assigned ID. The participants are compensated for their participation in the form of e-gift cards worth US $150 after the completion of 3 surveys and up to US $120 after the completion of the web-based modules if randomly assigned to the ES intervention.

### Design

This 5-year study comprises 2 phases. Phase 1 involves additional program development of 2 modules and a maintenance module. This phase of the work has been completed and will be described next. Phase 2 is a randomized clinical trial testing ES against Springboard Beyond Cancer on the web. In the last year, the team will complete the implementation planning to evaluate potential medical and community settings for implementing ES for future scale-up.

### Phase 1: Enhancements to the Original Empowered Survivor Content

#### Original Content

The development of original web-based intervention ES was described in the study by Manne et al [65]. Briefly, it had 4 modules: Introduction, Oral Care, Swallowing and Muscle Strength, and Long-term Follow-up Care and Detecting Lesions. These modules are listed in Table 1. Module 1, Introduction, orients participants and reviews modules. Module 2, Oral Care, reviews the causes, contains a symptom self-assessment, and provides information on how to manage dry mouth. Module 3, Swallowing and Muscle Strength, provides information on the symptoms of swallowing difficulty, methods to reduce swallowing difficulty, and methods to improve flexibility and range of motion in the head and neck. Module 4, Long-term Follow-up Care and Detecting Lesions, describes the importance of head and neck self-examinations; provides information on how to conduct a head and neck self-examination, signs to pay attention to, goal setting, and managing barriers to self-examination; and provides a link to a survivorship care plan website that includes follow-up information, assesses alcohol and tobacco use, and reviews the participant’s care team and recommendations. The original ES program had >20 interactive activities to engage participants and foster skill acquisition. An example is symptom assessment, which is followed by goal-setting feature. Participants rate symptom severity, frequency, concern about symptoms, and confidence in and importance of managing symptoms. Next, the participants select a goal related to the symptom; rate the importance of the goal; choose strategies to address the goal from a list of ideas; and rate benefits and barriers to achieving the goal, confidence in achieving the goal, and support needed to achieve the goal. This function is in modules 3 and 4.

**Table 1:** Empowered Survivor intervention modules.

Module			Goal		Activities
**Original modules**					
	1: Introduction	Orient participants and introduce team Review program modules and navigation Provide recommended modules Enhance engagement with survivor stories Introduce rationale, importance, and steps for self-management		Narrated introduction by oral surgeon to introduce self-management Discuss the importance of self-care and set expectations Sample goal-setting exercise	
	2: Swallowing and Strength	Understand the symptoms of swallowing difficulty Optimize swallowing ability Improve strength and mobility in head and shoulder Improve lymphedema management Facilitate personal goal setting		Self-assessment of swallowing difficulty, lymphedema, and flexibility Videos of swallowing and strengthening exercises Videos of exercises for lymphedema Goal setting, barriers, and strategies	
	3: Oral Care	Understand causes of dry mouth and what exacerbates it Assess dry mouth symptoms and severity Understand strategies to keep one’s mouth moist Assess nutritional needs Understand ways to improve nutrition Facilitate goal setting for dry mouth and nutrition		Videotaped introduction by surgeon Patient experiences with dry mouth Self-assessment of dry mouth symptoms Tips for managing dry mouth Food preparation for dry mouth Choose high caloric foods (quiz) Goal setting, barriers, and barriers	
	4: Long-term Follow-up Care	Understand how to do a self-examination Set goals and manage barriers for self-examination Develop a survivorship care plan that includes follow-up recommendations Create a personalized follow-up care plan Identify your care team and unmet care team needs and create a list of health care resources Learn strategies to communicate with oncology care team Assess current smoking and alcohol use Increase motivations to stop smoking and drinking Understand and manage other medical issues		Videotaped demonstrations of examinations Self-management tool to plan oral examination Get your survivorship care plan Unmet need assessment for survivorship care Who is your care team? Who does what? Do you have any communication concerns? Communicate with providers about survivorship Create a plan for managing comorbid illnesses Assess tobacco and alcohol use and treatment motivation Education about the health impact of continued use of alcohol Link to Drinkerscheckup and BetheMatch	
**New modules**					
	5: Calm and Connect	Improve the understanding of psychological concerns Enhance awareness about how feelings may affect self-care and that self-care of emotions is self-care Devise strategies to manage emotions and worries Assess and address special concerns for HPV^a^-associated oral cancer Unique concerns for young adult survivors Learn about survivors’ emotional reactions and coping		Rate worries and fears about cancer Distress assessment Rate confidence in the ability to manage worries and fears Videotaped stories of how survivors coped Link to meditation and mindfulness apps Choose coping strategy and goals Assessment of knowledge and worries about HPV-associated oral cancer	
	6: Maintaining	Continue working on skills and goals Learn new information about oral cancer Communicate with other participants		Select exercises and skills to move to “Favorites” Goals are migrated to this module and can be updated and reworked News posted regularly Message board	

^a^HPV: human papilloma virus.

#### Enhancements to the Existing Modules

Enhancements were selected based on feedback from the pilot study participants as well as the team’s experience with participants during the ES intervention. In module 1, a section containing information about self-care and goal setting to enhance self-care was added to familiarize the participants with the process. In module 2, the ability to set an email reminder to work on the oral care goal was included to optimize continued implementation. In module 3, because the participants liked the videotaped swallowing exercises and indicated that they would like to see more of them, additional exercises were developed with the team’s speech pathologist. The strengthening exercises were originally presented as illustrations but were very highly rated. To foster engagement, the team’s physical therapist developed videotaped demonstrations of exercises. Because lymphedema was mentioned as a survivorship concern in participant feedback from the pilot study, information about it and its management was added along with demonstrations of home exercises to manage lymphedema by the team’s physical therapist. Finally, additional information on how to add caloric content to food was added. In module 4, to bolster the existing content and address common concerns that were not addressed previously, additional resources were added for tobacco cessation, alcohol use, comorbid medical issues, managing hearing loss, and survivorship care planning. More content on interest in quitting tobacco use and the risks of continued use was added. For current smokers, a link to Becomeanex [103] was provided. If the person indicated a desire to continue smoking, material on the increased risk for cancer recurrence and mortality and reduced treatment benefits for smokers and an assessment of the readiness to quit were added by the team member MBS. As for alcohol use, if the participant indicated a desire to continue drinking, a link to Drinkerscheckup [104], an empirically based, web-based treatment, was provided [105]. As comorbid medical issues are common in this survivor population, a section was added on “other medical issues.” This section assesses comorbid medical problems, associated medications, and interest in improving the management of medical conditions and offers tailored input on the barriers to medication adherence based on an assessment of barriers. Finally, the team’s audiologist added additional material to address the management of hearing loss and tinnitus.

#### New Modules

A total of 2 new modules, which were originally planned but not developed, were created: module 5, Calm and Connect, and module 6, Maintenance. The goals of Calm and Connect are to describe common psychological concerns patients experience after treatment, increase the awareness of how cancer has affected the participant’s emotional well-being, assist the participant in developing useful methods to deal with emotions, provide free web-based resources on coping with stress, and discuss psychosocial aspects of having HPV-related oropharyngeal cancer. The introduction portion contains a video introduction by a psychologist portrayed by an actor and audiotaped narratives presenting patients’ emotional experiences. In the next 3 sections, the following coping skills are addressed: (1) managing stress with relaxation, (2) increasing the tolerance for upsetting emotions using acceptance-based strategies, and (3) talking it out with others. A fourth section was developed to address the unique concerns experienced by the survivors of HPV-associated cancers, including common worries (eg, transmission to others), managing questions from friends and family about HPV, and feeling embarrassed about HPV, and the unique experiences of young cancer survivors***.*** All sections include interactive activities. For example, the participants can select their key signs of stress, which activates a patient narrative about the signs of stress and narrated educational content. There are also audio relaxation exercises led by a psychologist portrayed by an actor, self-reflection exercises in which the participant types in responses, and links to outside resources such as web-based apps for meditation and mindfulness exercises (Headspace and Calm).

The goal of module 6, Maintenance, is to facilitate ongoing engagement in self-management, evaluate the progress on goals, provide opportunities for interaction with other study participants, and provide new information. This module has 4 features, namely (1) Favorites: the participants choose their favorite content and exercises from screens throughout the program. Across the ES program, 28 pages can be chosen as favorites. These pages are migrated to the Favorites section for future reference, and participants can log in directly to this content; (2) Goals: goals set by the participants in each module are automatically migrated to this section, where the participants can directly access, review, revise, and update them and set up email reminders about them to send to themselves; (3) News: articles of interest from science and medicine sources are posted by study staff on a regular basis; and (4) Message Board: the participants have access to the Message Board throughout their participation in this study. Every month or two, interesting news articles about oral cancer are placed on the board by the project team. When there is a post, the participants receive an email notification that a message has been posted. They can review the post and post messages at that time. They can also access the message board at any time.

Both enhancements to the existing content and new material were developed in collaboration with ITX, incorporated. ITX is a web-development company and the developer of the original ES intervention. Content was reviewed and implemented during a series of regular meetings between the study team and the ITX development team over a 6-month period. The videotaped and narrated content was created in collaboration with Mindful Designs.

#### Patient Advisory Board Feedback

The Patient Advisory Board consisted of 3 oral and oropharyngeal cancer survivors. The board members reviewed the Calm and Connect module after it was developed and provided feedback in a web-based meeting. Notes from the meeting were reviewed by the study team. No substantive content changes were made, other than those intended to fix grammatical and navigation issues.

**Figure 2:**
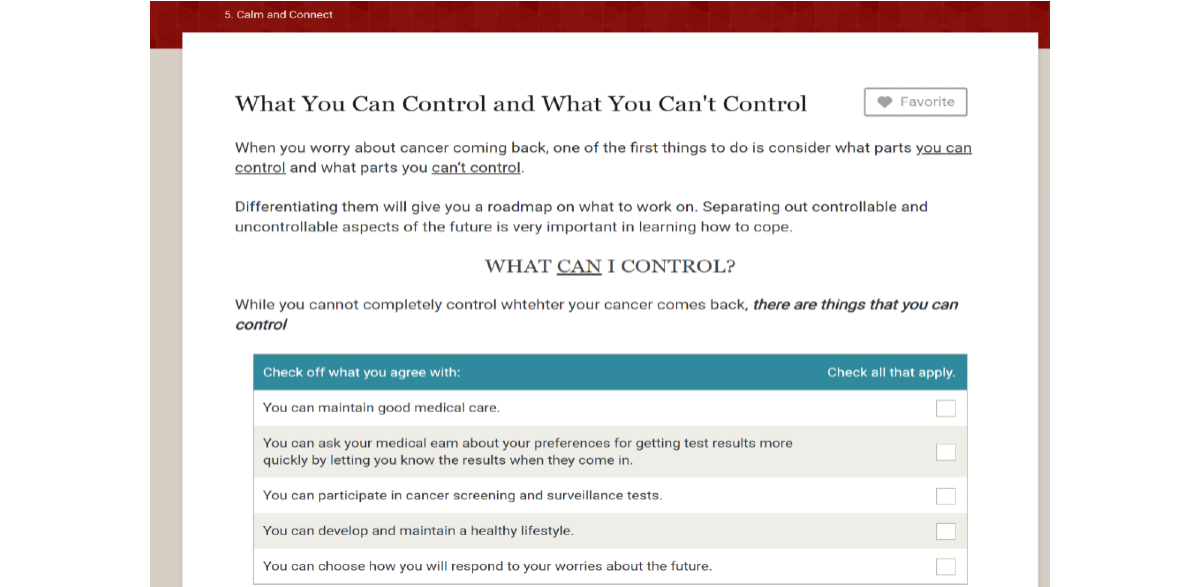
Sample page from Calm and Connect Empowered Survivor (1).

**Figure 3:**
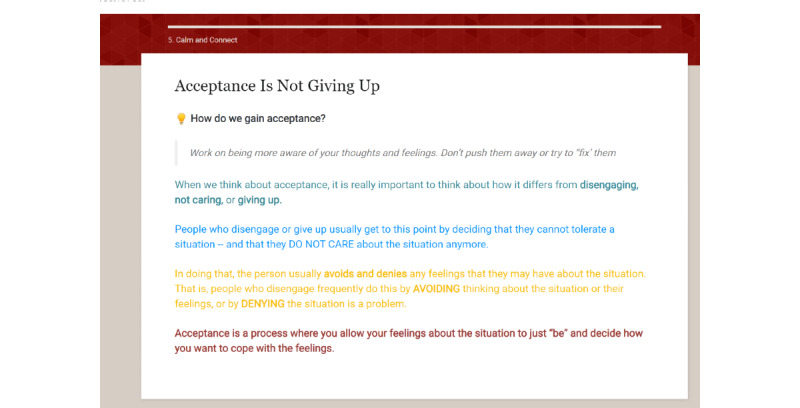
Sample page from Calm and Connect Empowered Survivor (2).

**Figure 4:**
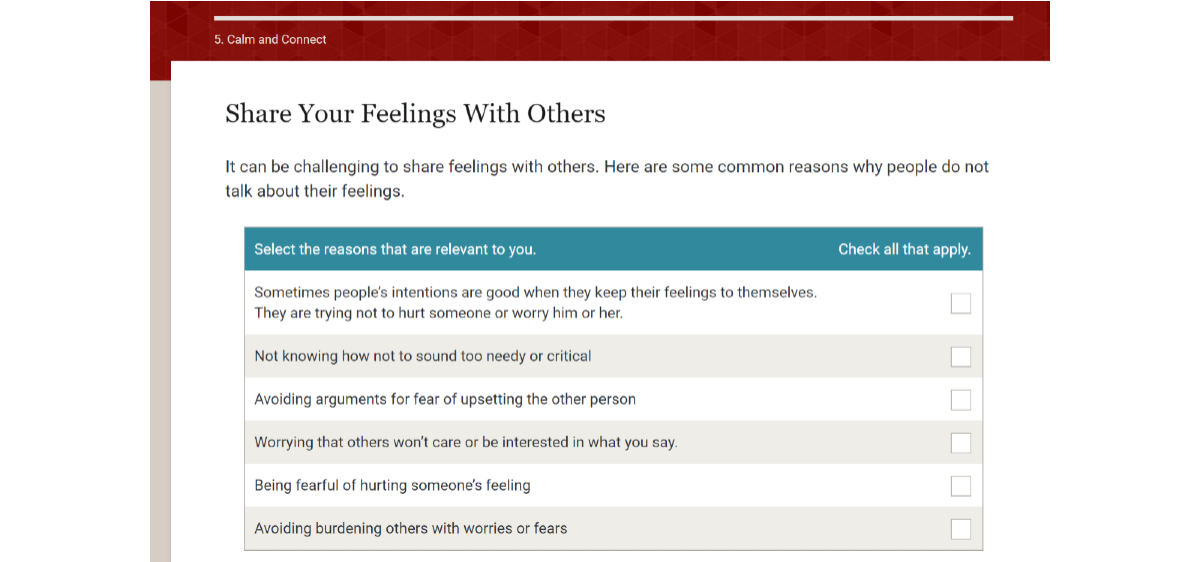
Sample page from Calm and Connect Empowered Survivor (3).

**Figure 5:**
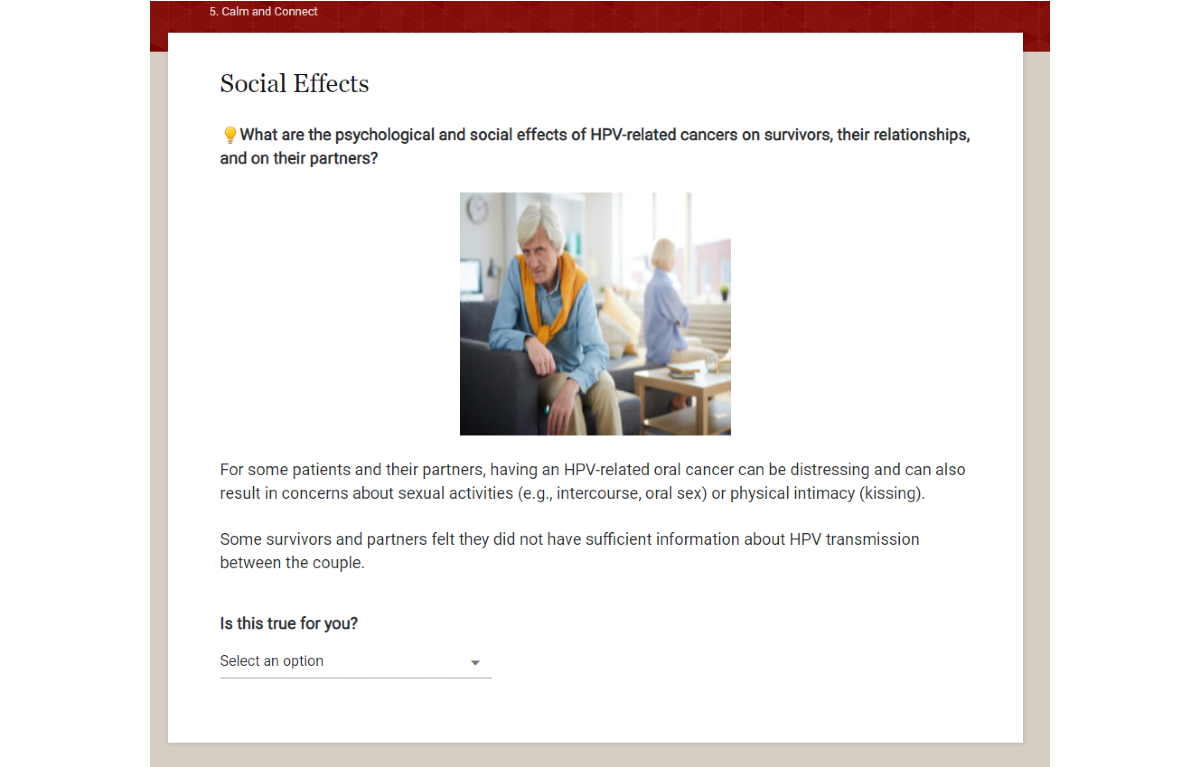
Sample page from Calm and Connect Empowered Survivor (4).

**Figure 6:**
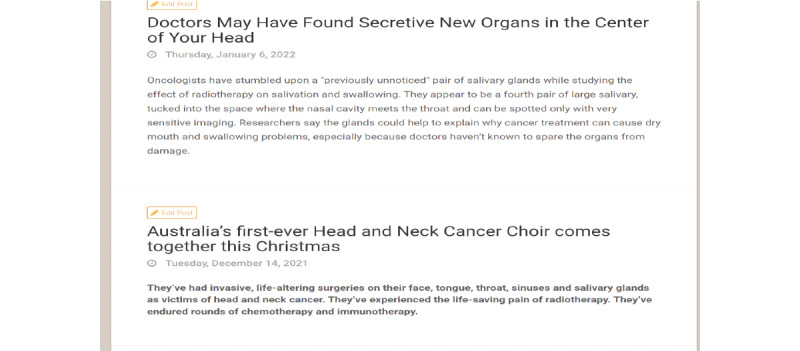
Sample Maintenance module screenshot (1).

**Figure 7:**
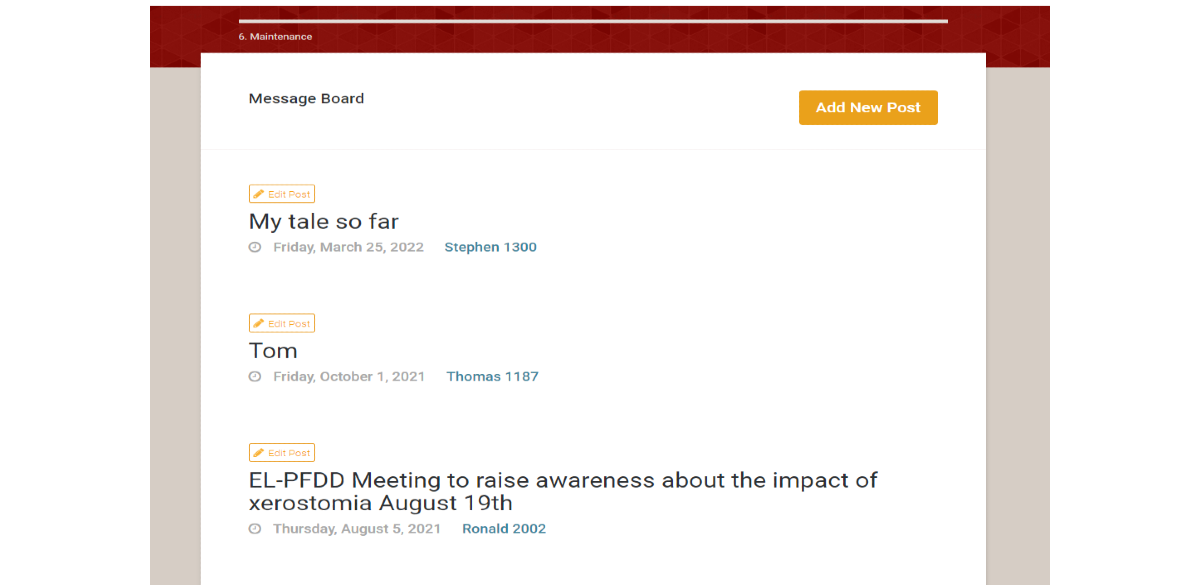
Sample Maintenance module screenshot (2).

**Figure 8:**
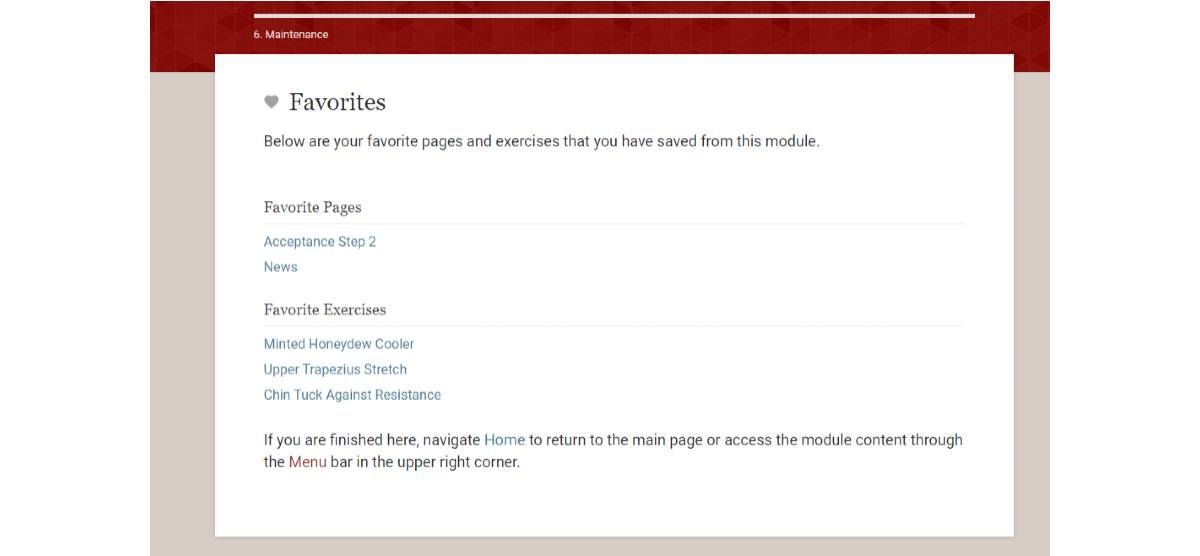
Sample Maintenance module screenshot (3).

After the new modules were finalized and enhancements were incorporated into the existing modules, the Patient Advisory Board reviewed ES. Board members were sent a link to ES and asked to review the content and note recommended changes and any feedback. Next, web-based 1:1 interviews were conducted to review content and collect feedback. The following key suggestions were given: several modules: correct navigation errors; module 1: provide more clarity with regard to navigating the menu bar, reduce expectations for goal setting by reminding participants that it may be easier to choose small goals, and add definitions of additional key terms; module 3: provide the ability to print the swallowing exercises; and module 5: add content to normalize the desire to get one’s life back to normal. The study team reviewed the interviews to select modifications. All of the listed changes were made. There were several suggested changes that were evaluated as less relevant to most survivors and were not incorporated. Content changes were implemented using ITX. The final content is shown in Table 2. The sample content from modules 6 and 7 are shown in Figures 2-8. There are more than 75 interactive activities in ES, including in-session exercises, live links, self-assessments, and video and audio narratives. Multimedia Appendix 1 [106] outlines these activities.

**Table 2:** Study measurement schema.

Measure		Screening	Baseline	Time 2	Time 3
**Covariates**					
	Date of birth	✓			
	Sex		✓		
	Marital status		✓		
	Race and ethnicity		✓		
	Education		✓		
	Insurance status		✓		
	Employment status				
	Regular primary care		✓		
	Financial hardship		✓		
	Tobacco use and history		✓		
	Alcohol use		✓		
	Date of diagnosis		✓		
	Treatment history		✓		
	Survivorship care summary		✓		
**Outcomes**					
	Self-efficacy	✓	✓	✓	✓
	Preparedness	✓	✓	✓	✓
	Head and neck QOL^a^	✓	✓	✓	✓
	Oral self-examinations	✓	✓	✓	✓
	Head and neck mobility exercises	✓	✓	✓	✓
**Mediators**					
	Planning		✓	✓	✓
	Activation		✓	✓	✓
	Information needs		✓	✓	✓
	Support needs		✓	✓	✓
	Fear of recurrence		✓	✓	✓
Intervention evaluation (treatment evaluation)				✓	
Qualitative data (structured interviews)					✓
Adverse event assessment (depressive symptoms)			✓	✓	✓

^a^QOL: quality of life.

### Phase 2: Randomized Controlled Trial

#### Design

**Figure 9:**
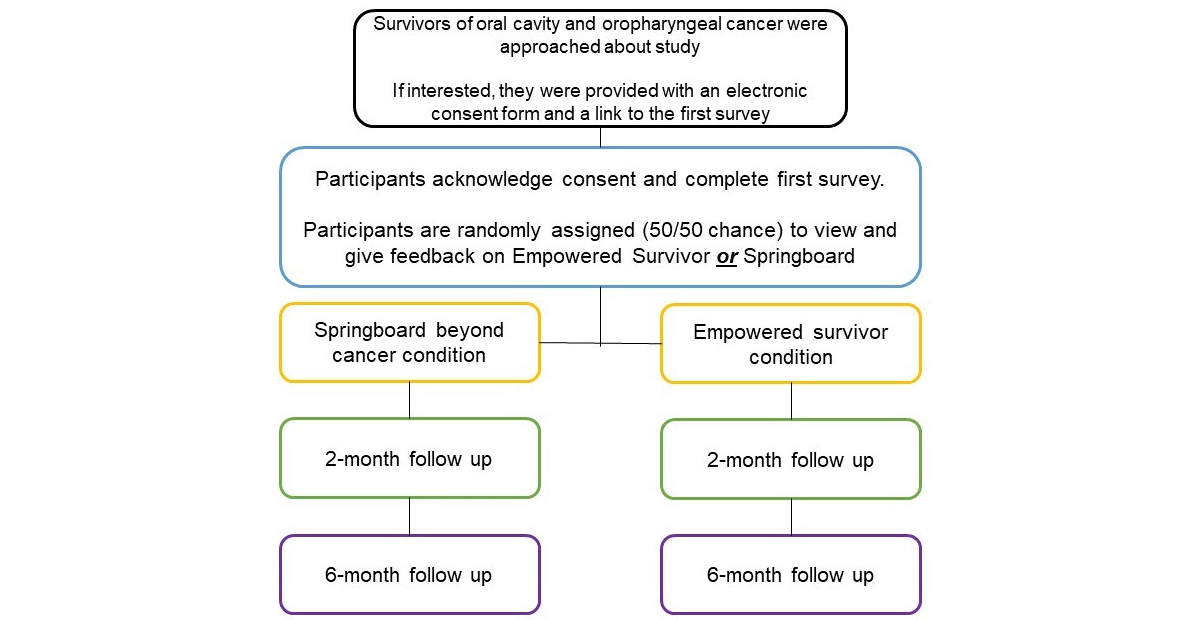
Study flowchart.

This is a prospective, randomized controlled trial to evaluate the effect of ES on confidence in managing self-care, preparedness, and health-related QOL and on the secondary outcomes of oral self-examination and engagement in head and neck strengthening exercises. This is evaluated in 2 groups with a total of 600 survivors (ES vs Springboard Beyond Cancer) and by 3 assessments (baseline, 2 months, and 6 months). The participants complete surveys at baseline and 2 months and 6 months after baseline. Figure 9 illustrates the participant flow. A free, publicly available general survivorship-focused web-based program, called Springboard Beyond Cancer, was selected for comparison based on a review of the literature on the design of behavioral interventions [98,107,108]. This review suggested that there are pros and cons to all comparison conditions and that selection should be guided by the phase of the research. We selected a comparison condition that had the same method of delivery (web-based delivery), same frequency of delivery, and similar topics.

#### Participants

The participants are recruited from 2 state registries and through clinic recruitment. The eligibility criteria are as follows: participants should (1) be aged >18 years, (2) be diagnosed with a first primary oral cavity or oropharyngeal cancer in the last 3 years, (3) be currently cancer free (but could have experienced a recurrence), (4) have computer access, and (5) be able to read English. Children are not included because the incidence of these cancers in children is exceedingly rare (0.2 cases/100,000 children).

Among the cancer registries, approximately 1600 patients are diagnosed with a first primary oral or oropharyngeal cancer per year. Our pilot study suggested that 26% of eligible patients will consent and complete the baseline survey. This enrollment rate is consistent with or higher than that of other intervention trials recruiting from cancer registries (18%-30%) [99,100]. Recruitment will be conducted for approximately 27 months. The sample size is based on the power to detect the effects on our primary outcomes. With 1600 patients available each year and 3 years of data collection, we have a large pool: given the 4800 total participants available and 26% acceptance, we have about 1104 potential participants. The power analysis suggests that the study requires 600 participants.

To achieve this, we include pictures of minority patients on our recruitment materials and oversample Black males in our registry recruitment. We have set a goal to ensure that of the total number of participants enrolled, 8% (n=48) are Black men.

#### Recruitment

Recruitment was primarily conducted using state cancer registries. Recruitment procedures differ between state registries. For the NJSCR, the registry confirms patient eligibility and, if eligible, sends a letter to the patient’s diagnosing physician, making the physician aware that their patient is eligible. If the NJSCR does not receive a disapproval from the physician within 2 weeks, the patient is sent a cover letter and an “agreement to contact” sheet. Potential participants are called by registry staff to discuss the study. The participants have the option of verbally consenting at that time. The participants who consent have their contact information and a copy of verbal consent forwarded to the members of the study staff, who call the patient. Patients deemed eligible are provided with a web-based consent and survey. The staff members contact patients weekly if they do not return the consent. Patients are considered passive refusers if they do not return a survey after repeated call attempts during days, nights, and weekends over a 3-week period after the consent is sent. For the Cancer Registry of Greater California and the Pennsylvania State Registry, the registry provides a list of patients and their contact information to the staff members. The staff members call patients, and those deemed eligible are provided with a web-based consent and survey, and the same procedures for enrollment as described above for the NJSCR are implemented. A maximum of 8 recruitment calls are allowed: 4 calls can be followed with a voicemail message, and 4 calls are counted as missed calls. Of these 8 contacts, 1 can consist of a text message. Once the electronic consent and survey are completed, the participants are automatically randomly assigned to the ES or Springboard condition using a data management system. Separate assignment schemas are set up for each registry by the study’s statistician.

Participants who do not complete the consent and baseline are contacted by phone or email on a weekly basis for 1 month to facilitate survey completion. The participants are automatically linked to one of the web-based interventions (ES or Springboard) as soon as they are randomized upon the completion of the baseline survey.

#### Interventions

##### Empowered Survivor

The ES program is described in Table 1, and Figures 2-9 provide examples of the content. The content is accessible via any device that is connected to the internet such as a tablet, an iPad, a phone, or a computer. If the participants experience issues in viewing information, they are contacted by phone to troubleshoot*.* The ES program can be completed in 1 sitting however, the orientation encourages the participants to complete ES over a longer period and emphasizes setting and implementing self-care goals and building self-management skills. A week after randomization, a team member contacts the participants to conduct a brief motivational discussion, the goals of which are to go over the content and benefits of the intervention, ask the participant whether they were able to log into the program, ask whether the participant whether they have questions, and offer to log in with the participant to review the Introduction module. The participants who do not log in by day 3 are sent a reminder email to log in, and the participants who do not log in by day 5 are sent a second reminder email to log in. When “News” or “Message Board” content is added, an email alert is automatically generated for the participants. The participants are provided a small incentive for completing the modules (US $20). The participants who do not log into the program within a month are entered into a lottery for a gift card worth US $20. These methods are recommended in systematic reviews and meta-analyses of factors important for revisits and continued use of internet interventions [101].

##### Springboard Beyond Cancer

**Figure 10:**
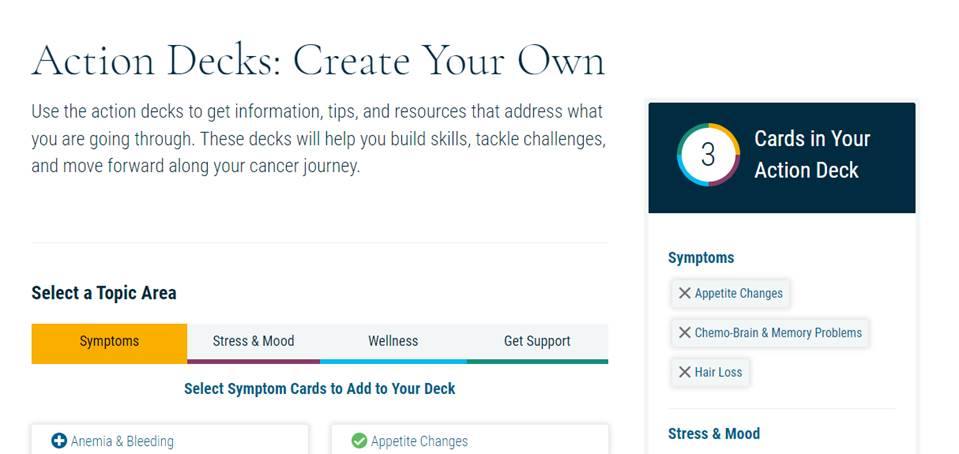
Sample page from Springboard Beyond Cancer (1).

**Figure 11:**
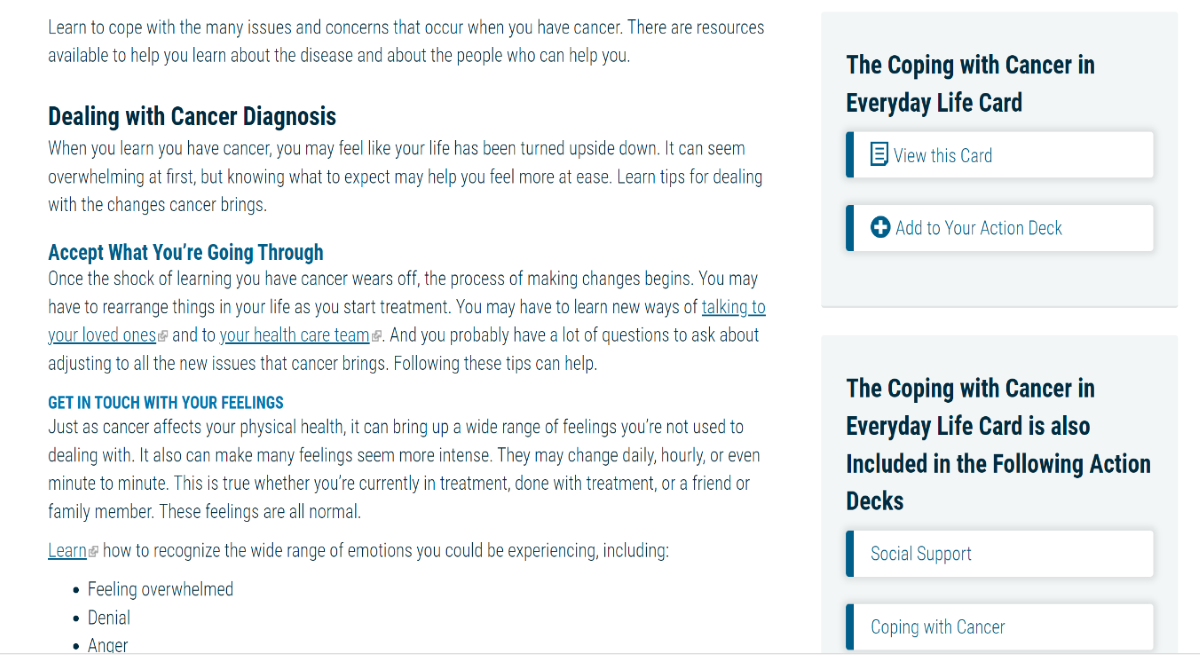
Sample page from Springboard Beyond Cancer (2).

**Figure 12:**
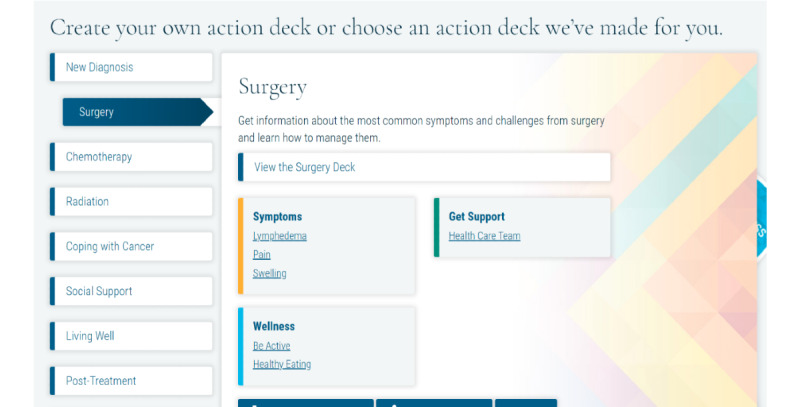
Sample page from Springboard Beyond Cancer (3).

**Figure 13:**
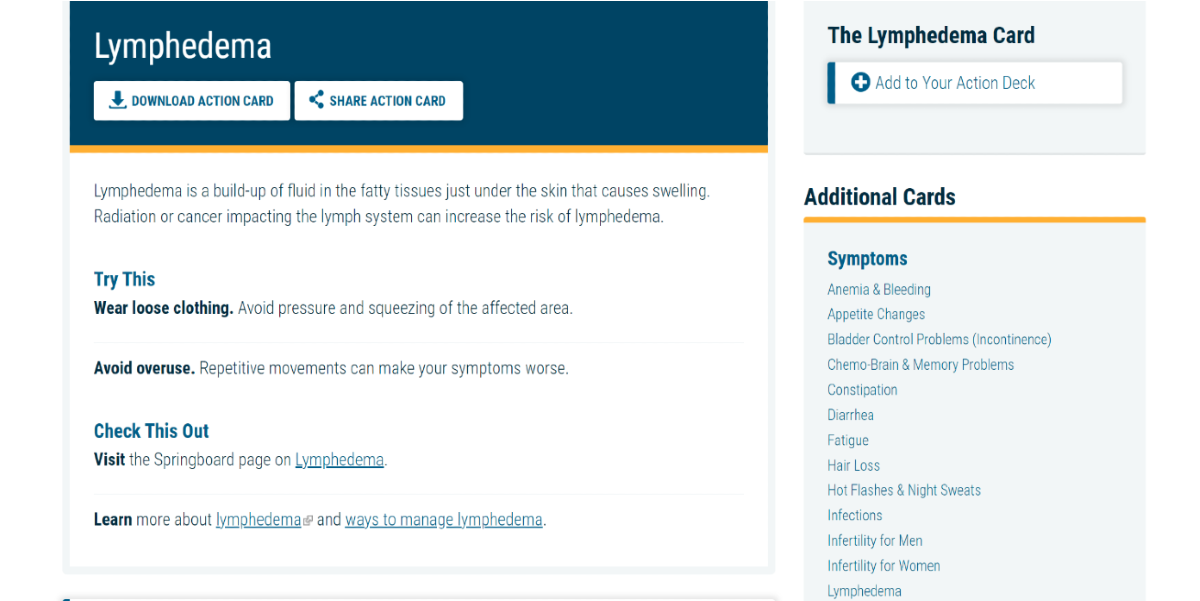
Sample page from Springboard Beyond Cancer (4).

Springboard Beyond Cancer is a self-management program developed by the ACS and the NCI. The program covers 4 main topics: Symptoms, Stress and Mood, Wellness, and Get Support. Each topic has several pages describing the topic and providing information on signs and symptoms, tips on how to manage the issue, and links to resources. The participants can move the tips to their Action Deck for future reference. Unfortunately, at the end of 2020, the NCI ended its contract with the ACS, and no longer hosted the Springboard website. The website was moved to an archive status [102], and the dynamic features of the site (eg, videotaped content) were disabled. After discussions with the NCI and ACS, an archived version of Springboard Beyond Cancer was shared with the study staff, and the NCI confirmed that the link would remain active throughout the study period. The team was able to provide the disabled videotaped content as a Springboard YouTube playlist [102]. This playlist houses all videos embedded in the Springboard website that were deactivated. In addition, the participants are provided with the ACS Survivorship: Before and after treatment website [109], which includes information on coping with cancer, maintaining a healthy lifestyle after treatment, and managing cancer as a chronic illness and survivorship resources (ie, care plans). The participants receive the Springboard YouTube link in the welcome email once they are randomized to the Springboard arm. A week later, they receive a second welcome email, which contains live links to ACS and Cancers survivor network. Sample pages are shown in Figures 10-13.

To facilitate engagement, within a week of sending the link to Springboard Beyond Cancer, the coordinator will reach out via phone to conduct a brief motivational discussion. The goals of this discussion are as follows: review program content and benefits, ask the participant whether they were able to log into the website, ask the participant whether they have questions, and offer to log in with the participant to review the Introduction. A week after the Springboard website is emailed to the participants, we will send another email with the ACS Survivorship website link. This email highlights sections of the website that the study team believes will be most useful for the participants. The coordinator manually sends an email or text to the participants 1 month after randomization, reminding them to return to the websites.

#### Survey Procedures

The participants complete electronic surveys via a web-based data management system (DatStat) at 3 time points: baseline and 2 months and 6 months after baseline. Table 2 presents the timing of the measures. At the 2- and 6-month time points, regardless of the intervention progress, the participants are sent an email with a link to the follow-up survey. To the participants who do not complete the survey within a week, 3 weekly email and phone call reminders will be sent. After 4 weeks, the participants who do not complete the survey will be considered noncompleters for that survey but will be sent a link to the next follow-up survey. The participants will receive a web-based gift certificate worth US $50 for completing each survey. Each survey takes approximately 30 minutes to complete.

#### Primary Outcome Measures

##### Oral Cancer Self-management Self-efficacy

This scale was developed for the pilot study [65] and consists of 22 items assessing participants’ confidence in dealing with 11 areas: management of dry mouth symptoms, dental care, proper nutrition, swallowing and speaking difficulties, neck and shoulder muscle flexibility and strength, follow-up care appointments, communication with providers, detecting lesions through self-examination, emotional responses, smoking, and alcohol use. One of the items assess the overall confidence in managing head and neck survivorship care. Ratings ranged from 1 (*not at all confident*) to 5 (*very confident*).

##### Preparedness

This 10-item scale assesses whether the information received about survivorship care was sufficient, helpful, comprehensive, and covered self-care tasks (1=*strongly disagree*; 5=strongly *agree*) [65,110-112]. Of the 10 items, 2 items assess how satisfied the cancer survivors are with the quantity of the information provided and the way in which the information was provided (1=*not at all satisfied* and 4=*very satisfied*).

##### Head and Neck Health–Related QOL

The European Organization for Research and Treatment of Cancer-Quality of Life Questionnaire-Head and Neck-43 (EORTC-HN-43) is the updated version of the EORTC H&N 35. This measure has strong convergent validity with EORTC H&N 35 [113-115]. The EORTC-HN-43 is the most widely used measure of head and neck QOL and has been rigorously and systematically validated in 18 countries.

#### Secondary Outcome Measures

##### Performance of Oral Self-examinations

The participants are asked whether they conducted a comprehensive examination of the inside of their mouth, the outside of their mouth (the face), and their neck to look for signs of oral cancer in the last month. The participants who report conducting an examination are asked whether they checked 11 different areas (eg, face, neck, inside of cheek, floor, and roof of mouth) and asked the degree to which they know what to look for when conducting an examination (1=*not at all*; 5=*completely*).

##### Engagement in Swallowing and Jaw and Neck Strengthening Exercises

The participants are asked whether they engaged in exercises to improve swallowing (tongue, jaw, Masako maneuver, Mendelsohn maneuver, and effortful swallow) in the last month (*yes/no*) and whether they engaged in exercises to improve or maintain muscle strength in the jaw and neck (jaw relaxation, turtle, and neck flexion and extension) in the last month (*yes/no*)*.*

#### Sociodemographic Measures

The participants report sex, age, education level, marital status, insurance status, race and ethnicity, income, and financial hardships in the last month (“Did you have adequate financial resources to meet the daily needs or you and your family?”).

#### Medical and Clinical Measures

At baseline, the time since diagnosis, whether the survivor had been diagnosed with a second cancer, recurrence status, treatments received (radiation, chemotherapy, and surgery), and comorbidities (Charlson index [116]) are reported by the participants. At all time points, oncology-related service use is reported by the participants. Tumor location and cancer stage are extracted from the tumor registry data. Tumors are characterized as oral, oropharyngeal, or salivary cancers. The participants also report the receipt of a survivorship care plan, their HPV tumor status, their current tobacco and alcohol use, and their current insurance status.

#### Mediator Measures

##### Action Planning

This 8-item measure assesses the degree to which a detailed plan has been made for self-care tasks (related to dry mouth, swallowing, muscle strength, detecting lesions, and long-term follow-up care) and how to manage interference in these plans (1=*strongly disagree*; 5=*strongly agree*) [94,117].

##### Patient Activation

This measure consists of 13 items measuring attitudes toward adopting an active role in care [118]. These items were adapted to assess head and neck cancer care. The following are a couple of sample items: “When all is said and done, I am the person who is responsible for managing my head and neck cancer care” and “I am confident that I can take actions that will help prevent or minimize some symptoms or problems associated with my head and neck cancer care” (1=*disagree strongly*; 5=*agree strongly)*.

##### Information Needs

This 23-item scale, adapted from the Health-Related Topics section of the Follow-up Care Use Among Survivors [119] and prior work, assesses [67,119] the desire for more information about oral cancer topics (1=*yes* and 0*=no*).

##### Support Needs

The Supportive Care Needs Survey [120] is a 34-item scale assessing physical, psychological, and health care system needs (1=N/A [not applicable]; 5=*high need*).

##### Concerns About Recurrence

Five items assess the worry about the recurrence of oral cancer (eg, “How much does the possibility that your head and neck cancer could recur upset you?”) [121]. An additional 27 items assess the degree to which worries about recurrence interfere with different life domains (eg, possibility of death, future treatment, femininity, sexuality, and body image).

#### Psychological Distress

The Patient Health Questionnaire-9 [122] is a self-report measure of depression that assesses key diagnostic criteria for depression based on the original PRIME-MD. It is a widely used measure and has demonstrated strong reliability and construct validity [123] in nonill populations as well as cancer populations [123]. Scores from 0 to 4 indicate no or mild depression, from 5 to 9 indicate mild depression, from 10 to 14 indicate moderate depression, from 15 to 19 indicate moderately severe depression, and from 20 to 27 indicate severe depression.

#### Treatment Evaluation

In both interventions, the following are assessed: (1) 17 items assess general features and content (use, helpfulness, learning something new, interesting, and valuable) and the degree to which the program improved confidence in managing symptoms and (2) 5 items assess website features (eg, for ES, survivor stories, goal setting, and discussion board and for Springboard, action card decks, symptoms page, stress and mood page, and wellness page). For ES, an additional 19 items assess the content of each module. Open-ended feedback is solicited.

#### Semistructured Interviews With ES Participants and Providers

A total of 30 participants from the ES condition participate in a brief web-based or phone interview. The survivors will be contacted after they complete the 6-month follow-up. The questions ask the participants about the best timing for the intervention (during or after treatment or another time), delivery schedule (all at once or one module at a time), best setting to offer the intervention (eg, dental visits or oncology care), and whether the participant would be willing to pay for the intervention.

In year 5 of this project, the team will conduct key informant interviews with 30 oral cancer providers (eg, oncologists and physical therapists) who have contact with this population. Questions will focus on the timing (eg, right after treatment), duration (weekly vs all at once), and settings (eg, during dental visits) that they feel would make ES most useful.

## Results

### Approach to Missing Data

To account for missing values owing to loss to follow-up, we will adapt the sensitivity analysis method proposed by Scharfstein [124] to estimate effects over ranges of assumptions about the relationship between dropout and response. To evaluate bias, we will examine the predictors of dropout at follow-up. We will evaluate demographic and medical factors, baseline alcohol use and smoking, and baseline self-efficacy as predictors.

We will use an intent-to-treat approach to analyze primary and secondary outcomes. Our approach to recruitment and retention minimizes missing data by contacting participants who do not complete sections of the survey and by incentivizing participation. Initial analyses will examine the characteristics of noncompleters. As recommended by Lang and Little [125], multiple imputations (using 50 imputed samples) will be used to impute missing values*.*

The treatment of covariates also warrants attention. Possible covariates include the demographic variables, objective risk variables, and medical variables of the survivors. Finally, to determine which covariates should be included in the analyses, preliminary analyses in which each outcome is predicted by the full set of qualifying covariates will be conducted. Only those covariates that significantly predict at least 1 outcome will be included in the analyses. Once the set of covariates is determined in this manner, the same set of covariates will be included in all analyses.

### Aim 1: Approach and Analysis Plan

#### Approach

The primary aim is to examine the relative impact of ES versus Springboard Beyond Cancer on our primary outcomes of self-efficacy in managing self-care, preparedness for managing survivorship, and health-related QOL at 6 months. We anticipate that 600 survivors will enroll (300 in each condition), and we conservatively estimate, based our ES pilot, that 80% (n=480) will complete the 1-month follow and 75% (n=450) will complete the 6-month follow-up. However, all available data will be included in the analyses.

#### Analysis

Multilevel modeling (MLM) will be used to examine whether there are significant differences between the ES and Springboard Beyond Cancer interventions over time. In our primary analysis, both time and treatment will be treated as categorical. This approach yields a main effect for treatment, a main effect for time, and an interaction between time and treatment. Covariates will include 3 effect-coded variables representing sex, race and ethnicity, marital status, education, age, tumor location, stage, time since diagnosis, whether the cancer recurred over the study period, whether the participant received a survivorship care plan, HPV status, and baseline tobacco and alcohol use. The MLM approach has the advantage that it does not exclude participants with missing data at some time points, as would be the case if a traditional ANOVA were used. Thus, this analysis used all available data.

#### Power and Sample Size

Power is calculated for the intent-to-treat effect sizes from the pilot study**,** assuming that 2-sided *z* tests are conducted at the *P*<.05 level. With an intent-to-treat analysis and a total sample of 600 participants, there is >99% power to detect significant differences between ES and Springboard Beyond Cancer in our primary outcomes of self-efficacy and preparedness for survivorship. There is 90% power to detect significant differences in health-related QOL at the 6-month follow-up.

### Aim 2: Approach and Analysis Plan

#### Approach

This aim evaluates the hypothesized mediators of the expected association between the intervention and outcomes and hypothesized moderators of the intervention effects. Mediators include action planning, information needs, support needs, patient activation, and the fear of recurrence. Moderators include race and ethnicity, education, receipt of a survivorship care plan, baseline self-efficacy, and preparedness. Figure 2 depicts the proposed mediational model in which the intervention impacts the survivors’ planning skills; reduces their information and support needs; and reduces their fear of recurrence, which ultimately impacts their self-efficacy, preparedness, and health-related QOL.

#### Analysis

Analyses will be conducted using 3 separate mediational models for the 3 primary outcomes. The indirect effects of ES on outcomes will be tested using changes in outcomes from baseline to 6 months as dependent variables, changes in the mediators from baseline to 2 months, and changes in mediators from baseline to 6 months. The SPSS macro (IBM Corp) will generate 5000 bootstrapped samples using random sampling with replacement to generate estimates and CIs around the indirect effects [126,127]. The mean of the 5000 indirect effect estimates based on the bootstrapped samples will be examined. Within a multiple mediator model, the indirect effect of each mediator is its unique effect on the outcome when controlling for the other mediators included in the model. We propose that ES will have stronger mediating effects than the Springboard Beyond Cancer intervention.

Treatment moderators will also be evaluated. Very little is known about which survivor subgroups benefit the most from self-management interventions. The available research suggests that survivors with fewer resources (eg, lower education) benefit more [128-132]. We will adopt a similar hypothesis and examine the role of low resources, people of color, lower education, not having received a written survivorship care plan, less baseline self-efficacy, and less baseline preparedness, in ES’s effects. For moderation analyses with continuous or dichotomous (eg, care plan receipt; White race or people of color) moderators, we will simplify the analysis by using MLM with a regression approach in which condition will be effect-coded (ES=1 and generic=−1) and time will be treated as a quantitative variable that was coded in months (0, 2, and 6) and then grand-mean centered. Moderator variables will be grand-mean centered (eg, baseline self-efficacy) or effect-coded (eg, care plan receipt). Moderation analyses will include the same set of covariates used in the primary analyses described for the primary aims.

#### Power and Sample Size

On the basis of the simulations and corrected bootstrap method outlined in Fritz and Mackinnon [130], there is at least a power of 0.85 to detect small effects (Cohen *d*=0.26) from both the independent variable to the mediators and the mediators to the dependent variable.

### Aim 3: Approach and Analysis Plan

#### Approach

This aim focuses on 3 recommended self-care tasks: oral self-examinations and the level of comprehensiveness of self-examinations, engagement in swallowing exercises, and engagement in head and neck mobility exercises.

#### Analysis

We will use the same approach as that outlined in the analysis of the primary aim.

#### Power and Sample Size

Assuming an intent-to-treat analysis and a total sample of 600 participants, we have 99% power to detect significant changes in oral self-examination and comprehensiveness of oral self-examination and 85% power to detect improvements in performance of swallowing exercises. Owing to low power, the performance of head and neck exercises is exploratory.

### Exploratory Aim: Approach and Analysis Plan

#### Approach

The broad implementation of a survivorship-focused intervention involves a community-based implementation. It is premature to broadly implement ES before determining its efficacy. However, as a first step in this process, we will conduct a process evaluation. First, we will use participant treatment evaluations and use data from ES. Systematic reviews have found that the average adherence to web-based health interventions is 50% to 53% [132,133]. ES use was higher in the pilot study in that 75% of the participants viewed 3 to 4 modules [65]. Most web-based interventions are designed to be used at least weekly for several weeks, but many participants access the interventions only once [132-134]. We will examine the predictors of ES use and evaluate the association of ES use with the primary outcomes. This analysis assists in identifying subgroups for targeting to improve the use of the intervention. Second, we interview a randomly selected subset of 30 ES participants to provide feedback upon the completion of the last follow-up. Third, we conduct key informant interviews with 30 oral cancer providers (eg, oncologists and physical therapists) who have contact with this population to identify potential settings for implementation.

#### Analysis

As for the participants, we record whether, how frequently, and for how long the participants logged into ES and how many modules are completed. In the analyses, we (1) examine whether these variables are associated with the primary outcomes, (2) evaluate the association of demographic factors (race and ethnicity, alcohol use, and tobacco use) with ES use, and (3) use an evaluation survey and web-based use data from ES.

For the survivor and provider interviews, interview data are transcribed. The analysis explores feedback on barriers to ES use in clinical practice and the preferred timing of ES (eg, should ES be introduced at a follow-up visit, in the waiting room, and by a navigator or health coach?). Analysis of interview transcriptions follow the “constant comparison” analytic approach [135], a method of explanation building in which the findings of an initial case are compared with a provisional category and revised as needed with new cases. This process is continued until an area of interest is fully explicated, reaching theoretical saturation [135]. Central to this process is “thematic” coding. We will develop an initial set of codes. For each code, we will develop ≥1 “secondary codes” that represent more specific aspects of the phenomenon. The schema organizes and assimilates data. The transcripts will be coded by at least 2 coders. Discrepancies will be resolved, and the process will be repeated until an acceptable level of intercoder reliability is achieved (using chance-corrected statistics such as κ for nominal data) [136]. Coded transcripts will be analyzed using the ATLAS.ti qualitative software (ATLAS.ti Scientific Software Development GmbH) [137].

This information will be used to create a website that serves as a storage house for all the study materials. On this website, the team will publish a free manual that details how to access ES and the methods to enhance ES use and contains information on hard-to-reach survivors and our publications and presentations.

### Data Safety and Monitoring

A serious adverse event (AE) is defined in this study in 2 ways. First, if the participant dies between baseline and follow-up, this is an AE. This situation will not be a study-related AE but will be reported to the Institutional Review Board. Second, if a participant endorses elevated depression at any of the 3 study survey time points, this is an AE. We monitor depression using the Patient Health Questionnaire-9 at each assessment time point. Scores in the moderate to severe range (defined as a score over 19) of depression indicate an AE. The procedure for handling AEs is that the study’s principal investigator will reach out to these participants by phone to assess distress, immediate need for intervention, interest in psychosocial services, insurance needs, and financial needs to determine the appropriate type of service. After assessing distress and support resource needs, our procedures include (1) a mailed list of national cancer patient resources (eg, the Cancer Support Community toll free hotline, Cancer Care support line, and the ACS support line); 2) a mailed list of cancer support groups at hospitals located close to the participant’s home, which will be prepared at the beginning of the study and updated regularly; and 3) a list of psychosocial providers within a 30-mile distance of the participant’s home, which will be provided by mail and over the phone. We monitor the participants who have endured distress at one time point during the course of the study using the follow-up surveys.

## Discussion

### Overview

The diagnosis and treatment of oral cavity and oropharyngeal cancers can adversely impact oral functioning and QOL. The management of disease- and treatment-related effects can be complex and involve oncology and rehabilitation experts to restore and prevent further deterioration of function. Survivors carry the primary responsibility for managing their follow-up and self-care. Adherence to this regimen is less than optimal, and negotiating the return to normal life can be challenging. Survivors experience decrements in QOL and desire information and support regarding long-term effects, recommended follow-up, oral symptoms that prompt contacting a physician, maintenance of nutrition, improvement of speech and swallowing, maintenance of oral health, and psychological concerns. In addition, survivors report low self-efficacy in managing their self-care regimen. Despite the substantial self-care needs, compromised QOL, low self-care self-efficacy, and low participation in self-care, there are only a few self-management interventions for this population. In our prior work, we developed and evaluated a self-guided web-based intervention to assist in self-management, called ES, which illustrated a promising impact in formative studies [65]. The goal of this study is to bolster the ES intervention by enhancing the existing content and adding 2 new modules focused on managing psychological concerns and maintaining oral cancer self-care routines and to assess the impact of the intervention on self-efficacy, preparedness, and QOL.

This study will provide important information regarding the impact of this self-management intervention when compared with a less-intensive intervention as well as information on the levels of engagement and predictors of engagement in ES. Further impact of this study lies in the information that will be gathered on its potential dissemination in the last phase of the study, which will identify the settings in which it may be implemented and incorporated into survivorship care.

### Limitations and Unanticipated Problems

The limitations of this study are the challenges related to recruitment into an intervention for cancer survivors, feasibility concerns associated with recruiting survivors via state cancer registries, reliance on self-report measures of oral self-examination and engagement in head and neck strengthening exercises, and well-described difficulties in engaging individuals in web-based interventions [138,139]. We leverage several strategies to enhance engagement, including lotteries for logging into the first module for those who do not log in after 2 weeks and incentives that will be provided after the completion of each module. In terms of feasibility, recruiting the participants via registries, although a way to increase diversity in a sample, can be challenging. Finally, although self-reported oral self-examinations and engagement in exercises are not the only self-care behaviors recommended for survivors, other self-care behaviors such as engagement in mouth care, changes in food intake to minimize swallowing difficulties, tobacco cessation, and alcohol use are less prevalent and less likely to apply to most survivors.

The findings of this study will provide important information on the efficacy of a web-based intervention on self-care among oral cancer survivors as well as initial information about the potential timing and settings for future scale-up. If efficacious, future research could evaluate potential implementation strategies to reach the survivors of oral cavity and oropharyngeal cancer.

## Appendix

Multimedia Appendix 1 Interactive components of Empowered Survivor.

## Abbreviations

ACS: American Cancer Society
AE: adverse event
EORTC-HN-43: European Organization for Research and Treatment of Cancer-Quality of Life Questionnaire-Head and Neck-43
ES: Empowered Survivor
HPV: human papilloma virus
MLM: multilevel modeling
NCI: National Cancer Institute
NJSCR: New Jersey State Cancer Registry
QOL: quality of life
