# Supplementary material for: Enhancing Self-care Among Oral Cancer Survivors: Protocol for the Empowered Survivor Trial
Source: JMIR Res Protoc. 2023 Jan 20;12:e39996. doi: 10.2196/39996 (PMC9898837; doi:10.2196/39996)
Supplement: Multimedia Appendix 1 [file resprot_v12i1e39996_app1.docx]

***Supplemental Table.*** Interactive Components of Empowered Survivor

| **Engagement Type** | **Topic** |
| --- | --- |
| **Module 1: Introduction** | |
| Video narrative | Physician welcome/introduction |
| Audio narrative | Narratives from three oral cancer survivors |
| Live link to resources | Learn more about goal setting |
| **Module 2: Oral Care** | |
| Video narrative | Physician introduction to Oral care |
| Audio narrative | Dry mouth symptoms survivor experience |
| Self-assessment | Rate your symptoms of dry mouth and severity |
| Self-assessment | Foods that influence your dry mouth symptoms |
| Create a printable list | Select and then print a shopping list |
| Live link to outside resources | Recipes: Click on them and will show recipe |
| Live link to outside resources | 5 live links to fee online or downloadable cookbooks |
| In-session exercise | Set an oral care goal to maintain dental hygiene/goal summary |
| Audio narrative | Story about survivors with dry mouth and how she managed it |
| **Module 3: Swallowing and Strength** | |
| Video narrative | Introduction video by speech pathologist |
| Self-assessment | Rate dysphagia symptoms experienced |
| Video narrative | Speech and swallowing exercising disclaimers physician video |
| Tailored care recommendation | Head and neck flexibility exercises tailored to cancer location and radiation location and level of experience |
| Live link to outside resources | Speech and swallowing exercise instructions |
| In-session exercise | Select benefits and barriers to doing swallowing exercises |
| In-session exercise | Goal setting for swallowing exercise |
| Video narrative | Closing video by speech pathologist |
| Video narrative | Physical therapy introduction video |
| Tailored care recommendation | Physical therapy treatment summary questions |
| Live link to outside resources | Neck and shoulder exercises |
| In-session exercise | Select benefits and barriers to doing exercises. |
| In session exercise | Set a goal for Head, neck and shoulder exercises. |
| Self-assessment | Does participant have head and neck lymphedema |
| Video Narrative | Closing summary by the physical therapist |
| Live link to outside resources | General Nutrition: Three live links to ACS, AICR, and new American plate guidelines for cancer prevention |
| Live link to outside resources | Phytonutrients in colorful fruits and vegetables hand out & to suggestion to increase calories for weight gain hand out. |
| In-session exercise | Set goal for managing your nutrition |
| In-session exercise | Select barriers to achieving your nutrition goal |
| Audio narrative | Survivor discussions of oral cancer treatment |
| **Module 4: Long-term Follow up Care** | |
| Video narrative | Physician introduction |
| Video narrative | Physician demonstrates oral self-exam |
| Live link to outside resources | Videos on how to conduct oral self-exams in key areas |
| Audio Narrative | Overcoming barriers to oral self-exams & what lesions look like |
| Video Narrative | Physician discusses what to do if you find something |
| In-session exercise | Set a goal for oral self-exam |
| Audio Narrative | Three oral cancer survivors describe experiences with follow up care |
| Self-assessment | Time since treatment was completed, recommended follow up, speech & swallowing, and dental check ups |
| In-session exercise | Create goal for general health behavior to improve |
| Live link to external resource | OncoLife^[136](#_ENREF_136)^ |
| Audio narrative | Oral cancer survivor’s survivorship care journey, |
| In-session exercise | Comorbidity assessment |
| Live link to external resource | Barriers to medication adherence |
| Self-assessment | Rate post treatment symptoms |
| Self-assessment | Last time had wellness visit with PCP |
| Self-assessment | Complete exercise questionnaire |
| Live link to external resource | American Council on Exercise |
| Self-assessment | Complete Hearing Handicap Questionnaire |
| Video Narrative | Audiologist intro video |
| In-session exercise | Symptoms of tinnitus with tinnitus impairment feedback |
| Live link to external resource | hearing and communication for available treatment information |
| Audio Narrative | how to understand your results after an audiological evaluation |
| Self-assessment | Receipt of radiation and chemotherapy |
| Self-assessment | Communication needs survey |
| Video Narrative | Audiologist describes effective communication with friends and family and how to seek a hearing assessment |
| Live link to external resource | Amplification and Implantable Technology |
| Self-assessment | Alcohol Use |
| Live link to external resources | National Institute of Alcohol Abuse and Alcoholism |
| Self-assessment | Tobacco use questionnaire |
| In-session exercise | Set goal for tobacco and alcohol use |
| Live link to external resource | BeAnEx Program and CDC national media campaign |
| **Module 5: Calm and Connect** | |
| Video Narrative | Psychologist describes the content in module |
| Self-assessment | Rate experienced emotions in the past month |
| Audio narrative | Four survivors describe their experiences |
| Self-assessment | Rate methods used to manage stress. |
| Self-assessment | Rate effects of stress on body, tension rating, relaxation setting. |
| Audio Narrative | Audiotaped focused breathing relaxation exercise |
| Audio Narrative | Progressive muscle relaxation exercise |
| Audio Narrative | Guided Imagery Exercise |
| Self-assessment | Relaxation progress questionnaire |
| Audio Narrative | Two survivors discuss their process of accepting cancer |
| Video Narrative | Two live links to Ted Talks about mindfulness and mediation exercises |
| Audio Narrative | Mindful Mediation exercise guided by psychologist |
| In- session exercise | Acceptance Exercise |
| Self-assessment | Rate degree to which participant shares feelings and reasons why they do not |
| In-session exercise | Rate current support needs and most important needs, how to ask |
| In session exercise | Rate current level of fear about cancer recurrence and top worries and select strategies to cope |
| Self-assessment | HPV related cancer & impact on intimate relationships after the diagnosis of HPV-related oropharyngeal cancer |
| Audio narrative | Physician closing narrative |
